# Factors associated with genital human papillomavirus infection among adult females in the United States, NHANES 2007–2010

**DOI:** 10.1186/1756-0500-7-544

**Published:** 2014-08-18

**Authors:** Runhua Shi, Srinivas Devarakonda, Lihong Liu, Hannah Taylor, Glenn Mills

**Affiliations:** Department of Medicine, Feist-Weiller Cancer Center, LSU Health Shreveport, Shreveport, LA USA

**Keywords:** Human Papillomavirus, Sexual behaviors, Prevalence Ratio, Survey

## Abstract

**Background:**

Patients with human papillomavirus (HPV) infection are at risk of developing cancer later in their life. Current research estimates the prevalence of genital HPV infection and explores the factors that are associated with the infection.

**Findings:**

The National Health and Nutrition Examination Survey 2007–2010 was used in this research study. The study population included females in the United States aged 18–59 years. The weighted prevalence of HPV infection was 41.9%. An estimated 59.4% of non-Hispanic black females had HPV infection. In a multivariate analysis, number of sexual partners, race, age, education level, marital status, income, smoking, and insurance status were associated with HPV infection. HPV infection was 5.77 times more likely for women with >11 sexual partners compared to women with 0–1 partners. Non-Hispanic black females were 1.87 times more likely to have HPV infection compared to non-Hispanic white females. Participants with only a high school degree had a 58% increased prevalence compared to college-educated women. Uninsured women had a 39% increased prevalence compared to those with insurance.

**Conclusion:**

This study found that 41.9% of U.S. females aged 18–59 years tested positive for genital HPV infection. We determined that individuals with more sexual partners, with a lower education level, with non-Hispanic black race, and with no insurance were the populations at greatest risk. It is necessary to continue monitoring the prevalence of this infection in the general population to provide a basis for effective treatment and prevention in the target populations.

## Findings

Genital human papillomavirus (HPV) is the most common sexually transmitted infection in the United States. HPV includes a group of more than 150 related viruses. Many of these viruses can be easily spread through direct skin contact during sexual intercourse [1]. More than 50% of individuals engaging in sexual activities are infected with at least one type of HPV in their lifetime. An estimated 42.5% of U.S. females aged 14–59 years had a genital HPV infection in 2003–2006 [2]. Some patients with HPV infection can be restored back to health, while infection progresses to cancer in others [3].

More than 40 HPV types can infect the genital areas of men and women [4]. These types can be classified as high-risk, probable high-risk, low-risk, and undetermined risk for the development of cervical cancer. Low-risk types (HPV 6, 11) are mostly associated with genital warts. High-risk types (HPV 16, 18) can contribute to precancerous lesions, low-grade cervical intraepithelial lesions and high-grade cervical intraepithelial lesions, as well as anogenital cancers [5]. HPV infection is a major cause of cervical cancer [6], and 11,818 women in the U.S. were diagnosed with cervical cancer in 2010 [7].

The primary goals of our current research are to identify the potential factors associated with HPV infection and to estimate the prevalence of infection for U.S. females from 2007 to 2010. Eventually, we aim to help create programs targeting high-prevalence populations to prevent HPV infection and lower their risk of getting cervical cancer.

## Methods and materials

Data was obtained from the National Health and Nutrition Examination Survey (NHANES) 2007–2010 [8]. The survey data includes information about demographic and socioeconomic status, mental health, and dental health, as well as physiological and laboratory measurements. The survey interviews about 12,000 people biannually.

### Survey design and population

The NHANES used a multistage probability sample design to select the participants. Consenting participants completed a household interview followed by a physical examination and interviews at a Mobile Examination Center (MEC). Non-Hispanic (NH) black and low income groups were oversampled in the NHANES to allow for an accurate statistical estimation in these population groups [9]. The protocol was approved by the National Center for Health Statistics (NCHS) institutional review board. Further information regarding study design and methods for oversampling is available on the NHANES website [8].

From 2007 to 2010, 10,010 females of all ages were interviewed. The combined unweighted household interview response rate for that period was 78.9%; the examination response rate was 76.3%. All females aged 18–59 years (n = 4242) who visited the MEC were asked to self-collect a cervicovaginal swab sample. Out of all samples collected, 3738 (88.1%) were reported as positive or negative and were used in the final analysis. Combining the data for years 2007 through 2010 was justified. There was no significant difference in HPV prevalence between 2007–2008 and 2009–2010 for all but 5 of 37 HPV types, as detected by the Linear Array assay (data not shown).

### Demographic and behavioral data

Demographic information, including gender, age, race, education, marital status, and country of birth, was obtained from all participants during the household interviews. The poverty index was calculated according to the U.S. Census definition. This method divides total family income by the poverty threshold after adjusting for family size at the time of the interview.

Sexual history information, including if the participant had ever had sex and the age of first sexual experience, was self-reported by participants using an audio computer-assisted self-interview. Respondents who reported having sex (described as vaginal, oral, or anal) were asked additional questions about their lifetime sexual history and about any sexual encounters in the prior 12 months. These additional questions addressed number of sexual partners, sexual orientation, and condom usage.

### Specimen collection and processing, laboratory methods

As described by Dunne et al. [10], self-collected cervicovaginal swab samples were obtained from female participants aged 18–59 who had an examination in the MEC. Swabs were given to the NHANES personnel, stored at room temperature, and mailed within 1 week to the Centers for Disease Control and Prevention (CDC) laboratory. There, they were kept at 4°C and extracted within 1 month of collection.

Multiplex polymerase chain reaction (PCR) was used for detection of 37 HPV types within the *Alphapapillomavirus* genera. Samples were reported as HPV positive if any of the 37 HPV deoxyribonucleic acid (DNA) types were detected, including high-risk (16, 18, 26, 31, 33, 35, 39, 45, 51, 52, 53, 56, 58, 59, 66, 68, 73, 82) and low-risk (6, 11, 40, 42, 54, 55, 61, 62, 64, 67, 69, 70, 71, 72, 81, 82 subtype IS39, 83, 84, 89) types. If the strips were positive for any of the types, the sample was coded as positive. If the strips were negative for all of the types and beta-globin was detected, the sample was coded as negative. If there was no beta-globin present in the sample and no HPV type was detected, the sample was coded as inadequate [8, 11–14].

### Statistical analysis

We estimated the overall prevalence of infection for any HPV type with respect to sociodemographic and sexual behavioral characteristics. Due to the complexity of the design, all estimates were measured using 4-year MEC sample weights provided by NCHS to account for the unequal probabilities of selection and adjustment for nonresponse. The weighting methodology has been described previously [10]. Taylor series linearization was used to estimate variance in a complex cluster survey design [15]. Confidence intervals (CIs) were calculated using a logit transformation with the standard error of the logit prevalence based on the delta method and applying SUDAAN estimated standard errors [16].

The Wald χ2 statistic was used to assess bivariate association between HPV and sociodemographic or behavioral characteristics. No adjustments were made to the p*-*values for multiple comparisons. An unconditional logistic regression model was used to determine associations between genital HPV infection and these factors. Variables for adjustment in multivariate logistic regression models were selected based on bivariate associations with p-values <0.2. Goodness of fit for the final step of the model was assessed using the Hosmer-Lemeshow Satterthwaite adjusted *F* test.

SAS software version 9.4 for Windows (SAS Institute Inc. Gary, North Carolina) and SAS callable SUDAAN 11.0.1 [17] were used for the statistical analyses. Two-sided p-values less than 0.05 were considered statistically significant.

## Results

**Figure 1:**
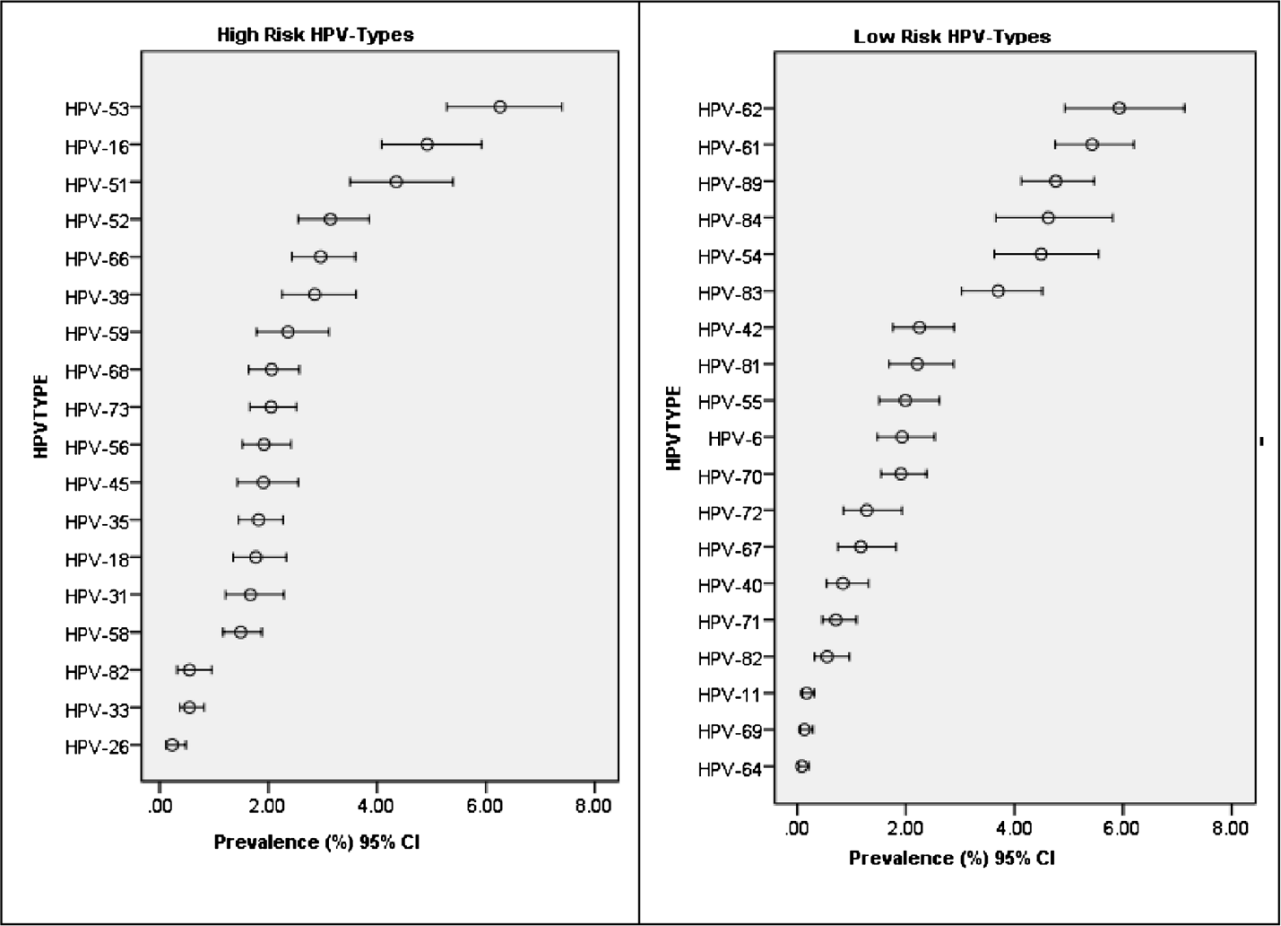
**HPV types and species by oncogenic risk category. Left panel**, High-Risk HPV Types. **Right panel**, Low-Risk HPV Types.

HPV types and species by oncogenic risk category are presented in Figure 1. In the NHANES 2007–2010, there were 3738 females aged 18 to 59 years with an HPV evaluation result in the final analysis. The 37 HPV types were grouped as high-risk (Figure 1 Left Panel) or low-risk (Figure 1 Right Panel). The prevalence of high-risk HPV types 33 and 58 was less than 2%; HPV strains 16 and 18 had a prevalence of 4.92% and 1.77%, respectively. HPV strain 53 had the highest prevalence of 6.26% in the high-risk HPV category, and HPV strain 62 had the highest prevalence of 5.93% in the low-risk category.

In a univariate analysis (Table 1), the overall weighted prevalence of HPV infection was 41.9%. An estimated 59.4% NH black females and 38.7% of NH white females, the lowest prevalence, were positive for HPV. There was a statistically significant difference between subjects of different races. The Prevalence Ratio for NH black females was 2.3 times that of NH white females.

**Table 1:** **Weighted prevalence of HPV infection among female participants ages 18–59 by sociodemographic characteristics NHANES 2007-2010**

		Sample size		%	95% CI of % HPV+				95% CI of PR	
		HPV+	HPV-	HPV+	Lower	Upper	p-value#	PR	Lower	Upper
	**All**	**1716**	**2022**	**41.9**	**39.6**	**44.2**				
Race	NH White	676	916	38.7	36.0	41.5	<0.0001	1.00		
	NH Black	436	299	59.4	54.4	64.2		2.32	1.90	2.83
	Mex. Amer.	321	457	41.9	38.1	45.7		1.14	0.97	1.34
	Other	283	350	40.7	35.3	46.4		1.09	0.85	1.38
Age	18-24	383	287	56.1	51.4	60.7	<0.0001	1.00		
	25-29	222	192	50.8	45.9	55.6		0.81	0.64	1.01
	30-34	189	244	40.1	33.8	46.7	<0.001*	0.52	0.39	0.70
	35-39	203	267	38.3	33.6	43.2		0.49	0.36	0.65
	40-44	182	295	34.5	30.4	38.9		0.41	0.33	0.53
	45-49	227	256	44.4	39.2	49.8		0.63	0.48	0.81
	50-54	172	266	33.4	27.1	40.4		0.39	0.29	0.53
	55-59	138	215	34.0	27.3	41.4		0.40	0.29	0.56
Education	> = College	243	480	31.4	27.4	35.6	<0.0001	1.00		
	<High School	486	484	48.0	43.6	52.3		2.02	1.54	2.63
	High School	430	418	47.5	42.2	52.8	<0.001*	1.98	1.54	2.54
	Some College	554	637	43.5	39.3	47.9		1.69	1.34	2.13
Marital Status	Married	562	1154	29.4	26.8	32.2	<0.0001	1.00		
	Wid/Div/Sep	393	278	58.3	53.4	63.0		2.96	2.08	4.20
	Never married	457	322	55.6	50.0	61.0		2.10	1.50	2.94
	Living W Partner	188	157	55.2	47.1	63.1		3.00	2.46	3.66
	Missing	116	111	46.7	39.3	54.3		3.35	2.68	4.19
Ratio of family income to poverty	PIR:> = 350%	343	620	33.3	29.4	37.4	<0.0001	1.00		
	PIR:130-349%	541	655	43.0	39.2	47.0	<0.001*	1.42	1.19	1.71
	PIR <130%	691	581	55.3	51.9	58.6		2.33	1.90	2.84
Country of Birth	US	1338	1437	42.7	40.0	45.4	0.1095	1.00		
	Mexico	161	281	35.6	30.7	40.8		0.743	0.598	0.923
	Other	217	304	39.1	33.0	45.6		0.863	0.651	1.142

#P-value: Chi-Square Test; *P-value (p-values for CMH trend analysis, without missing categories), PR: Prevalence Ratio.

There was a lower trend of HPV infection with increased age, higher education level, and decreased family poverty level. There was a bimodal pattern in the prevalence of HPV for age, with a high HPV prevalence of 56.1% in the 18–24 age group and with a second peak in the 40–44 age group. HPV prevalence was 48% for women with less than a high school education, whereas it was 31.4% for those with a college education. Participants living in a family with an income-to-poverty ratio (PIR) of <130% showed a 55.3% prevalence of HPV infection. In terms of marital status, prevalence was lowest (29.4%) for married women and highest (>47%) for women classified as single, divorced, or never married.

The association of HPV infection with behavioral factors is presented in Table 2. Females who smoked <100 cigarettes in their lifetime (Prevalence Ratio [PR] = 0.52) were less likely to have HPV infection. Females who did not have health insurance (PR = 1.9) or a routine place to receive health care (PR =1.39) were more likely to have HPV infection.

**Figure 2:**
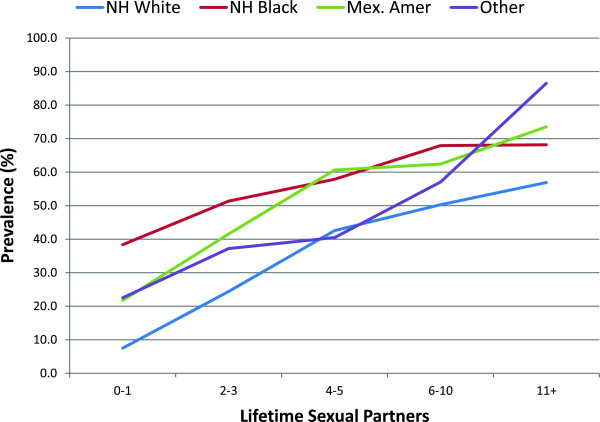
**Weighted prevalence of genital HPV among female adult participants aged 18–59 by lifetime sexual partners and race.**

Females who reported never having sex were 71% less likely to have HPV infection compared to females who reported having sex. The prevalence of HPV infection increased 89% for females having sex before 16 years of age compared to after 16. There was a trend of increasing HPV infection in women with a higher number of sexual partners in their lifetime (or yearly). The prevalence of infection was 8.9 times higher for women with >11 sexual partners in their lifetime compared to women with 0–1 partners. This trend was noticed among all races. Overall, NH black females showed the highest prevalence of HPV infection regardless of their number of partners (Figure 2). Compared to women who used a condom during sex, those who did not use a condom had twice the prevalence of HPV infection. Lastly, women who identified themselves as being ‘lesbian’ or ‘bisexual’ had a 72% increased prevalence of HPV infection compared to those who identified as ‘straight.’

**Table 2:** **Weighted prevalence of HPV among female participants ages 18–59 by behavioral factors**

		Sample size		%	95% CI of % HPV+		p-value#		95% CI	
		HPV+	HPV-	HPV+	Lower	Upper		PR	Lower	Upper
Smoked ≥ 100 cigarettes in life	Yes	763	608	51.2	47.3	55.1	<.0001	1.00		
	No	838	1304	35.3	32.5	38.3		0.52	0.44	0.62
	Missing	115	110	45.7	38.3	53.3		0.80	0.56	1.15
Drug Use	Yes	857	713	49.0	45.4	52.7	<.0001	1.00		
	No	642	1066	33.2	30.0	36.4		0.79	0.58	1.06
	Missing	217	243	43.0	37.3	48.9		0.52	0.43	0.62
Covered by health insurance	Yes	1157	1494	38.5	35.6	41.5	<.0001	1.00		
	No	557	524	54.4	51.1	57.7		1.90	1.58	2.29
Routine place for health care	Yes	1443	1736	41.0	38.5	43.5	0.0057	1.00		
	No	273	285	49.0	43.6	54.5		1.39	1.10	1.75
Ever had vaginal, anal, or oral sex	Yes	1467	1659	42.6	40.2	45.1	<.0001	1.00		
	No	33	118	17.6	11.9	25.4		0.29	0.19	0.43
	Missing	216	245	43.1	37.3	49.1		1.02	0.78	1.33
How old when first had sex	> = 16 years	489	357	54.5	50.0	58.8	<.0001	1.00		
	<16 years	976	1296	38.8	36.2	41.4		1.89	1.59	2.24
	Missing	251	369	37.1	32.2	42.3		0.93	0.74	1.18
#male sex partners lifetime	0-1	139	514	14.8	12.0	18.1	<.0001 <.0001*	1.00		
	2-3	264	429	31.2	27.2	35.5		2.61	2.05	3.32
	4-5	298	289	45.8	39.6	52.1		4.87	3.51	6.75
	6-10	422	306	54.3	49.5	59.1		6.85	5.40	8.69
	11+	334	180	60.7	55.5	65.6		8.90	6.41	12.35
	Missing	259	304	42.3	37.4	47.4		4.23	3.08	5.81
#male sex partners yearly	0	194	338	33.7	28.8	38.9	<.0001 <0.001*	1.00		
	1	929	1283	37.3	34.9	39.7		1.09	0.93	1.27
	2	178	57	74.8	66.8	81.5		4.10	2.96	5.70
	3+	159	32	85.2	77.0	90.9		6.89	4.61	10.30
	Missing	256	312	41.3	36.6	46.3		1.28	1.06	1.55
#times had sex without condom/year	Never	288	356	40.9	35.7	46.3	<.0001 <0 011*	1.00		
	<half of the time	185	123	58.6	51.6	65.2		1.10	0.88	1.38
	half of the time	99	57	63.5	54.6	71.5		2.25	1.59	3.19
	>half of the time	120	83	56.3	48.8	63.5		2.76	2.00	3.82
	Always	546	716	38.6	35.3	42.0		2.05	1.48	2.84
	Missing	478	687	37.1	33.7	40.6		0.94	0.79	1.12
Describe sexual orientation	Heterosexual or straight	1355	1639	40.9	38.4	43.6	0.0034	1.00		
	Homosexual/lesbian/Bisexual/Other	133	111	54.4	46.2	62.4		1.72	1.26	2.36
	missing	228	272	42.5	37.2	47.9		1.07	0.83	1.37

#P-value: Chi-Square Test; *P-value (p-values for CMH trend analysis, without missing categories), PR: Prevalence Ratio.

In a multivariate logistic regression analysis, adjusting for other factors, the number of lifetime sexual partners had a significant association with HPV infection. HPV infection was 5.77 times more likely for women with >11 partners compared to women with 0–1 partners. NH black females were 1.87 times more likely to have an HPV infection compared to NH white females. Age was negatively associated with the prevalence of HPV, except for an increased peak in the 44–49 age group. Having a college degree and being married were also associated with lower HPV prevalence. Family income-to-poverty ratio, a habit of smoking cigarettes, and insurance status remained significant factors associated with HPV infection.

When the results were adjusted for other factors, country of birth, recreational drug usage, ever having sex, age at first sexual encounter, condom usage during sex, and sexual orientation were no longer associated with HPV infection.

## Discussion and conclusion

From the NHANES 2007–2010 data set, we found that the overall prevalence of genital HPV infection was 41.9% (95% CI: 39.6-44.2%) in females aged 18–59 in the United States. We compared this result with two previous NHANES analyses observing data for females in the United States. Our estimate was similar to the result of the first study, which found a 42.5% (95% CI: 40.3-44.7%) prevalence for females aged 14–59 in the 2003–2006 data set [2]. The confidence intervals overlapped, indicating no statistical difference between these two estimations. Our estimate was higher than the result of the second study, which found a prevalence of 26.8% [10] among females, aged 14–59 in the 2003–2004 data set. This discrepancy could be due to a change in the laboratory methods to detect HPV DNA resulting in increased sensitivity of the test [2, 18, 19].

In our study, there was a bimodal prevalence of genital HPV for age, consistent with the same two previous NHANES studies [2, 10]. This may indicate increased sexual activity in the 18–24 age group [20, 21] causing the transmission of HPV by sexual contact. The reason for the increased prevalence in the 45–49 age group is unclear. We speculate that it may be due to an increased incidence [22], a difference in sexual behaviors across birth cohorts [23], or a change in marital status.

HPV infection was associated with certain racial and ethnic groups. NH black females had an increased prevalence even when controlling for factors such as number of sexual partners in their lifetime (Figure 2) or other factors (Table 3). Interestingly, even in the group with 0–1 sexual partners, NH black females had twice the prevalence of HPV infection compared to other racial and ethnic groups. This indicates that number of sexual partners is not the only factor that impacts the prevalence of HPV infection.

**Table 3:** **Multivariate logistic regression analysis of HPV infection with sociodemographic and behavioral factors**

			95% CI	
		Odds ratio	Lower	Upper
Race	NH White	1.00		
	Mex. Amer.	1.50	1.16	1.95
	NH Black	1.87	1.45	2.42
	Other	1.13	0.84	1.52
Age	18-24	1.00		
	25-29	0.81	0.57	1.15
	30-34	0.52	0.36	0.76
	35-39	0.52	0.36	0.75
	40-44	0.46	0.33	0.63
	44-49	0.76	0.53	1.09
	50-54	0.48	0.34	0.68
	55-59	0.59	0.39	0.87
Education	> = College	1.00		
	<High School	1.36	0.95	1.95
	High School	1.58	1.22	2.05
	Some College	1.13	0.89	1.43
Marital Status	Married	1.00		
	Living W Partner	1.47	1.02	2.10
	Never Married	1.74	1.34	2.25
	Wid/Div/Sep	2.27	1.72	3.00
	Missing	29.66	1.93	456.93
Ratio of family income to poverty	>350%	1.00		
	<130%	1.38	1.11	1.71
	130-349%	1.06	0.87	1.29
Country of Birth	US	1.00		
	Mexico	0.81	0.57	1.15
	Other	1.35	0.94	1.94
Smoked at least 100 cigarettes in life	> = 100 Cig/life	1.00		
	<100 Cig/life	0.78	0.62	0.99
	Missing	0.03	0.00	0.40
Drug Use	Yes	1.00		
	No	1.06	0.85	1.31
	Missing	1.15	0.36	3.73
Covered by health insurance	Yes	1.00		
	No	1.39	1.12	1.73
Routine place to go for healthcare	Yes	1.00		
	No	0.95	0.72	1.26
Ever had vaginal, anal, or oral sex	Yes	1.00		
	No	0.58	0.15	2.17
	Missing	0.96	0.18	5.17
Age when first had Sex	> = 16 years.	1.00		
	<16 years	0.94	0.79	1.12
	Missing	0.34	0.09	1.25
#male sex partners/lifetime	0-1	1.00		
	2-3	1.99	1.46	2.72
	4-5	3.69	2.62	5.21
	6-10	4.87	3.54	6.71
	11+	5.77	3.64	9.15
	Missing	15.21	5.12	45.18
#male sex partners/year	0	1.00		
	1	0.97	0.64	1.49
	2	2.17	1.10	4.28
	3+	3.12	1.47	6.65
	Missing	0.55	0.22	1.36
#times had sex without condom/ year	Never	1.00		
	<Half of the time	1.18	0.80	1.74
	Half of the time	1.23	0.74	2.07
	>Half of the time	0.97	0.64	1.47
	Always	1.06	0.78	1.44
	Missing	0.68	0.43	1.07
Describe sexual orientation	Heterosexual/straight	1.00		
	Homosexual/lesbian/bisexual/other	1.24	0.92	1.67
	Missing	1.43	0.74	2.74

#Prevalence Ratio (PR). If the 95% Confidence Interval of the PR does not contain the value 1.0, the association between the HPV infection and factor is statistically significant (p<0.05).

Some studies have shown an inconsistent association between education level and prevalence [24, 25]. However, in others, education level was negatively associated with HPV infection [2, 10]. In our study, with increased education, the prevalence of HPV infection decreased. We speculate that this trend could be connected to factors such as increased awareness of HPV or adoption of safe sexual practices. Safe practices might include, but are not limited to, regular usage of condoms and limiting the number of sexual partners. Therefore, health education interventions could be introduced to reduce the HPV prevalence by increasing awareness, encouraging safe sex [26], teaching about the routes of HPV transmission, and promoting the use of the HPV vaccine [27].

The number of sexual partners played a statistically significant role in HPV infection. This finding mirrors those seen in other studies [2, 10]. We also found an association of health insurance status with HPV infection. A meta-analysis showed that cost of vaccination and lack of insurance coverage are barriers that prevent women from obtaining the vaccine [28]. Individuals with private health insurance are more likely to hear about the HPV vaccine and three times more likely to get the vaccine compared to uninsured patients or those with public insurance plans [29]. In addition, poverty and having smoked <100 cigarettes in their lifetime were associated with HPV infection.

In conclusion, 41.9% of U.S. females aged 18–59 years tested positive for genital HPV infection. This study found that an increased number of sexual partners, a lower level of education, non-Hispanic black race, and a lack of insurance were factors of concern with HPV infection. Continuing to monitor the prevalence of HPV in the general population can establish a basis for possible interventions focusing on at-risk groups.

### Availability of supporting data

All data used in this research can be downloaded from the following website: http://www.cdc.gov/nchs/nhanes/nhanes_questionnaires.htm.

### Ethics statement

Use of data from the NHANES 2007–2010 is approved by the National Center for Health Statistics (NCHS) Research Ethics Review Board (ERB) Approval for NHANES 2009–2010 (Continuation of Protocol #2005-06), NHANES 2007–2008 (Continuation of Protocol #2005-06) and NHANES 2005–2006 (Protocol #2005-06).

## Abbreviations

HPV: Human papillomavirus
NH: Non-hispanic
CDC: Centers for Disease Control and Prevention
NHANES: National Health and Nutrition Examination Survey
MEC: Mobile Examination Center
NCHS: National Center for Health Statistics
PCR: Polymerase chain reaction
DNA: Deoxyribonucleic acid
PIR: Income-to-poverty ratio
PR: Prevalence ratio
SAS: Statistical analysis system
SUDAAN: Survey data analysis.
